# Neuropilin1 silencing impairs the proliferation and migration of cells in pancreatic cancer

**DOI:** 10.1002/jcla.23394

**Published:** 2020-05-30

**Authors:** Li‐Hong He, Yong‐Lin He, Wen‐Hang Zuo, Yue Kang, Huan Xue, Ling‐Yun Wang, Yun‐Liang Zhang, Yong Meng

**Affiliations:** ^1^ The First Hospital of Lanzhou University Lanzhou China; ^2^ The First School of Clinical Medicine of Lanzhou University Lanzhou University Lanzhou China; ^3^ School of Clinical Medicine Southwest Medical University Luzhou China; ^4^ Department of General surgery The First Affiliated Hospital of Shantou University Medical College Shantou China

**Keywords:** cell migration, cell proliferation, lentiviral interference, Neuropilin1, pancreatic cancer

## Abstract

**Background:**

Neuropilin1 (NRP1) participates in cancer cell proliferation, migration, and metastasis as a multifunctional co‐receptor by interacting with multiple signal pathways, but few studies have addressed the precise function of NRP1 in pancreatic cancer (PACA) cells. We aimed to study whether NRP1 gene silencing involved in the proliferation and migration of PACA cells in vitro.

**Methods:**

A lentiviral vector expressing NRP1 shRNA was constructed and transfected into human PACA cells (CFPAC‐1 and PANC‐1). The expression of NRP1 protein and mRNA was detected by Western blot and quantitative real‐time polymerase chain reaction (qRT‐PCR) assay, respectively. CCK‐8 assay, wound healing assay, and transwell assay were conducted to examine the effect of NRP1 silencing on cells proliferation and migration capability.

**Results:**

Results of qRT‐PCR and Western blot showed successfully established, stably transfected shRNA‐NRP1 cells in PACA cells. The proliferation capacity of PACA cells in NRP1 shRNA group was lower significantly than that in the negative control (NC) group (*P* < .05). The invasion and migration capability of PACA cells in NRP1 shRNA group was lower significantly than that in the NC group (*P* < .01).

**Conclusions:**

NRP1‐shRNA lentiviral interference vectors can effectively decrease NRP1 gene expression in PACA cells, thereby inhibiting cells proliferation and migration, which provides a basis for finding a valuable therapeutic target for PACA therapy.

**Abbreviations**
ECL: Enhanced chemiluminescence
EDTA: Ethylenediaminetetraacetic acid
EMT: Epithelial mesenchymal transition
FBS: Fetal bovine serum
NRP1: Neuropilins1
NSCLC: Non‐small cell lung cancer
RNAi: RNA interference
shRNA: Short hairpin RNA
TGF‐β: Transforming growth factor β
VEGF: Vascular endothelial growth factor

## 1 INTRODUCTION

Pancreatic cancer (PACA) is one of the most aggressive human cancer types, accounting for about 2% of all malignant tumors. Owing to the special biological characteristics of the pancreas and its anatomical location, the cancer is associated with high malignancy rate, low early diagnosis rate, and poor prognosis.^1^ Globally, the overall 5‐year survival rate of PACA patients is <5%. Although radical surgery is potentially the only optimal curative treatment for PACA, a vast majority of patients (80%) have already lost this chance at the time of diagnosis.^2^, ^3^, ^4^ In combination with the improvement of standard of living and population aging, we are faced with an increasingly serious challenge of PACA.

NRP1 was first found as an axon adhesion protein in the nervous system of frogs, encoding a single type I transmembrane glycoprotein of molecular weight 120‐130 kDa.^5^ Later research found that NRP1 gene expression is widespread over a variety of cells and tissues, such as endothelial cells, heart, liver, lung, kidney, pancreas, and skeletal muscle.^6^ It is a non‐tyrosine kinase transmembrane glycoprotein located on the cell membrane, acting as co‐receptors for members of the vascular endothelial growth factor (VEGF) family and for secreted Semaphorin3A. In addition, NRP1 also plays a vital role in cardiovascular system, nervous system, immune system, and other aspects of tumor invasion and metastasis through interaction with various ligands, which can promote angiogenesis, neural development, cytoskeleton remodeling, initial immune response, and occurrence and development of tumors.^7^, ^8^, ^9^ There is increasing evidence had indicated that higher expression of NRP1 associated with diverse malignant tumors of human, including hepatocellular carcinoma,^10^ breast cancer,^11^ gastric cancer,^12^ and prostatic cancer.^13^ NRP1 can promote tumor angiogenesis, cell proliferation, and cell migration, through a variety of mechanisms that play a vital role in the progression of cancer.^14^, ^15^, ^16^ The role of NRP1 in malignant tumors of human has become a research hotspot in recent years.

However, to our knowledge, the expression level, biological function, and underlying mechanisms of NRP1 in PACA remain poorly understood.^17^ The important role of NRP1 in many malignant tumors has prompted us to study whether NRP1 is a potential therapeutic target for PACA. In this research, we observed CFPAC‐1 and PANC‐1 cells in vitro for cell proliferation, invasion, and migration in human PACA after NRP1 knockdown by lentivirus‐mediated RNA interference. Our study provides an experimental basis for gene‐targeted treatment of PACA.

## 2 MATERIALS AND METHODS

### 2.1 Human PACA cell lines

CFPAC‐1 and PANC‐1 cells were purchased from the Shanghai Cell Bank and cultured under standard condition containing PMRI 1640 medium (Gibco) supplemented with 1% Penicillin‐Streptomycin (100 IU/mL; Gibco) and 10% fetal bovine serum (Gibco) with 5% CO_2_ at 37°C. When the cell density was consistent with the density indicated in the previous article, lentivirus transfection and cell function assays were carried out.^18^ All operations were carried out in the biosafety cabinet after ultraviolet sterilization.

### 2.2 Lentivirus vector and transfection

Based on the results of bioinformatics, we selected three short hairpin RNA (shRNA) targeting human NRP1 sequence (sh1 NRP1: 5′‐AAAGCCCCGGGTACCTTACAT‐3′; sh2 NRP1: 5′‐AACACCTAGTGGAGTGATAAA‐3′; sh3 NRP1: 5′‐AACAGCCTTGAATGCACTTAT‐3′). The sh1 NRP1 was finally selected for subsequent experiments. The lentivirus vector used in this experiment was produced and provided by a company (Hanyin Biotechnology Limited Company). More concretely, two single‐stranded DNA were designed and synthesized: 5′‐gatccAAGCCCCGGGTACCTTACATTTCAAGAGAATGTAAGGTACCCGGGGCTTTTTTTTTg‐3′ and 5′‐aattcAAAAAAAAAGCCCCGGGTACCTTATTCTCTTGAAATGTAAGGTACCCGGGGCTTTg‐3′. Then, double‐stranded DNA, formed by annealing the single‐strands, was connected to the linearized vector and was transformed into competent cells. The plasmid was extracted, enzyme digested and sequenced, and the lentivirus was packed into 293T cells. According to the instructions provided by the company, CFPAC‐1 and PANC‐1 cells were transfected transiently with the above lentivirus. We have a scrambled/non‐targeting control (sense, 5′‐GGCTCTAGAAAAGCCTATGC‐3′), which is named NC group, and we set cell lines transfected with the empty vector as a control and it was named Blank group.

### 2.3 Quantitative real‐time polymerase chain reaction (qRT‐PCR)

In this work, qRT‐PCR was performed according to the previously reported method.^18^, ^19^ NRP1 and GAPDH primers were synthesized and purchased from Sangon Biotech: NRP1 sense, 5′‐ATCACGTGCAGCTCAAGTGG‐3′, and antisense, 5′‐TCATGCAGTGGGCAGAGTTC‐3′; GAPDH sense, 5′‐AAGGTGAAGGTCGGAGTCAAC‐3′, and antisense, 5′‐GGGGTCATTGATGGCAACAATA‐3′. The experiments were repeated in triplicate. All experimental details were given in Supporting information.

### 2.4 Protein extraction and Western blot (WB) analysis

In this study, protein extraction and WB assays were carried out according to the previous method.^18^ Briefly, the protein extraction reagents and enhanced chemiluminescence kit were purchased from Thermo, Israel. The NRP1 antibody was purchased from Abcam company. Finally, the relative proteins were quantified by Image‐Pro Plus 6.0 (Media Cybernetics). All experimental details were given in Supporting information.

### 2.5 Cell proliferation, invasion, and migration assays

To determine cell proliferation activity, we performed CCK‐8 assay, experiments in 6 replicates. To determine cell invasion and cell migration capability, we performed transwell assay and wound healing assay, experiments in 3 replicates. The above assays were carried out according to the previous method.^18^, ^20^ All experimental details were given in Supporting information.

### 2.6 Statistical analysis

All data were analyzed using SPSS 20.0 software (IBM Corp.), and were summarized and presented as the mean ± SD. Student's *t* test and ANOVA (Tukey's multiple comparison test) were used to compare statistical significance in the various groups. *P* < .05 was considered statistically significant difference.

## 3 RESULTS

### 3.1 Detection of NRP1 mRNA expression in the target cells after transfection with qRT‐PCR

**Figure 1:**
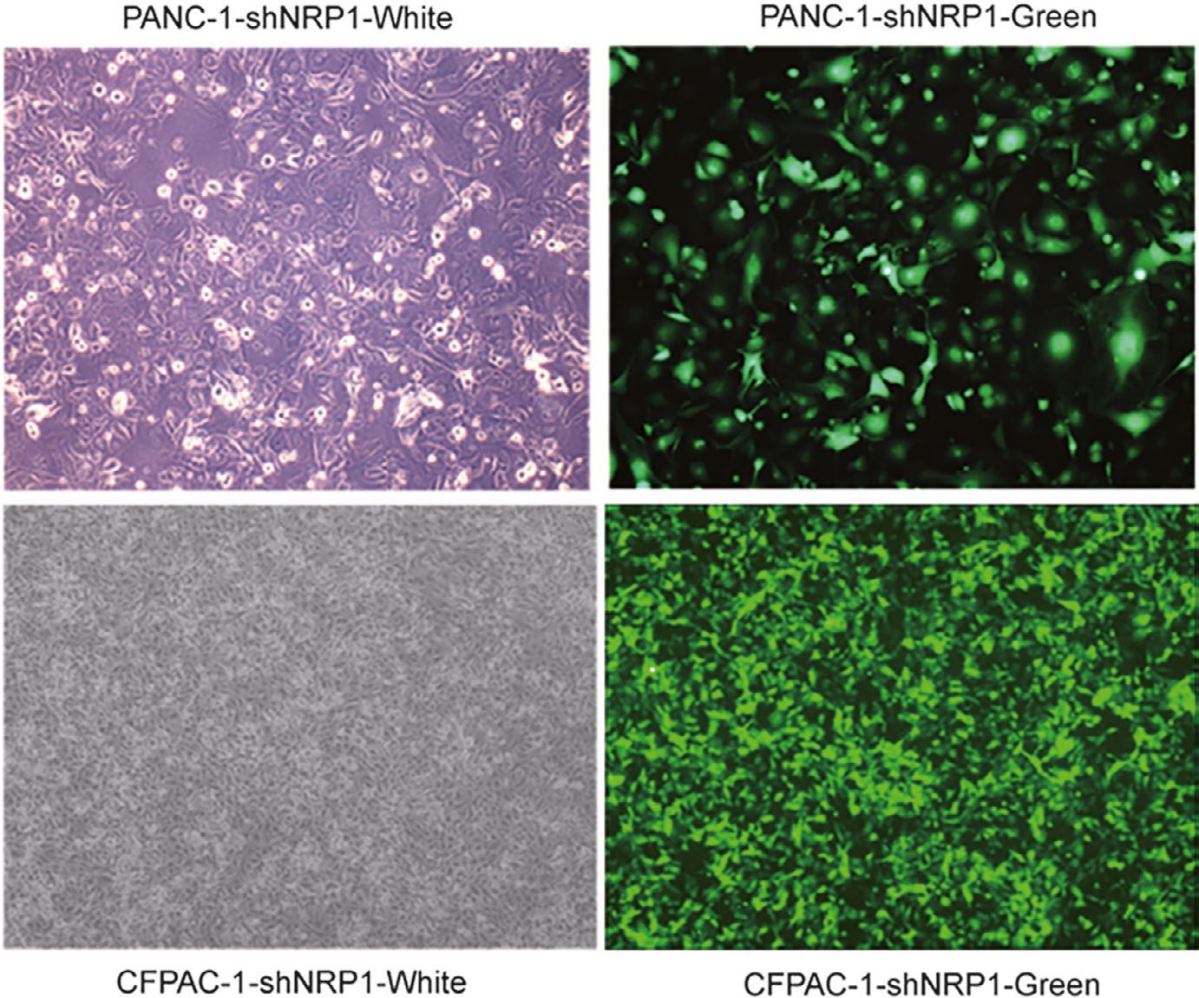
Expression of green fluorescent protein in negative shRNA group and shNRP1 virus transfection group

In order to detect the transfection efficiency of sh1 NRP1, sh2 NRP1, and sh3 NRP1, on the third day after transfection, mRNA was extracted from all types of cells. Then, the expression level of NRP1 mRNA was detected by qRT‐PCR. Results showed that NRP1 mRNA level in three shRNA targeting groups reduced significantly compared to that in the negative control (NC) group (*P* < .01; Figure 1). Meanwhile, as shown in the Figure 1, the sh1 NRP1 interference worked best.

### 3.2 Determination of transfection efficiency

**Figure 2:**
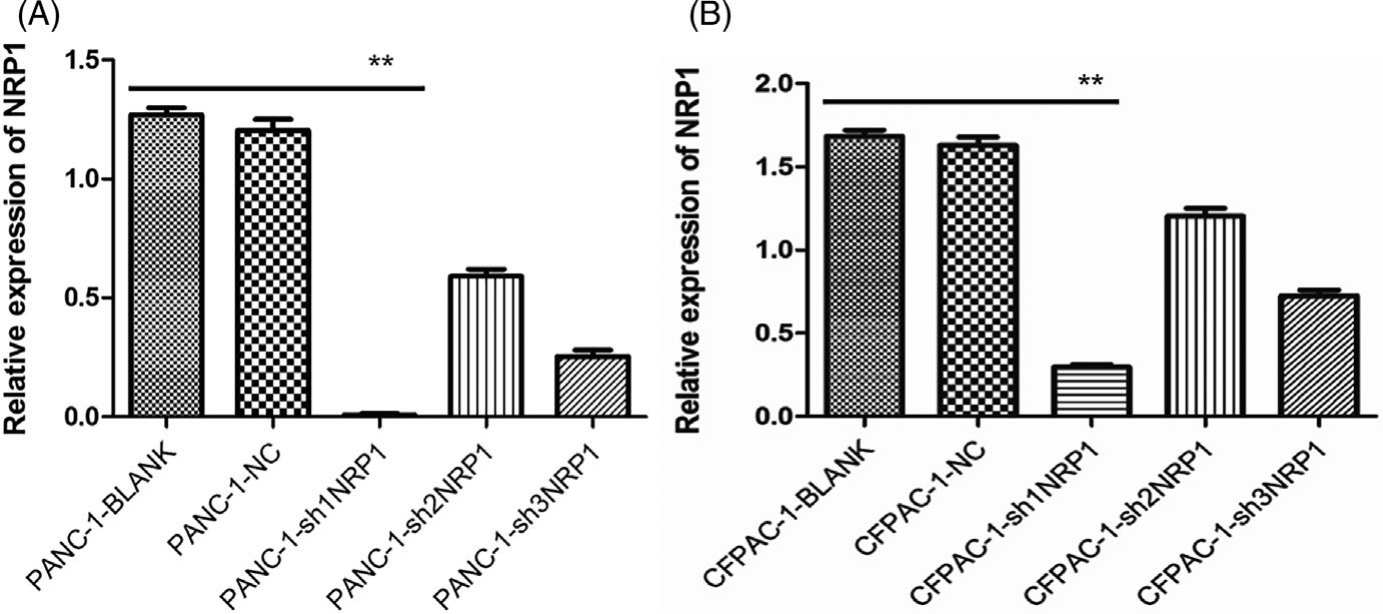
Expression of NRP1 mRNA in (A) PANC‐1 and (B) CFPAC‐1 cells after transfection, as detected by qRT‐PCR

**Figure 3:**
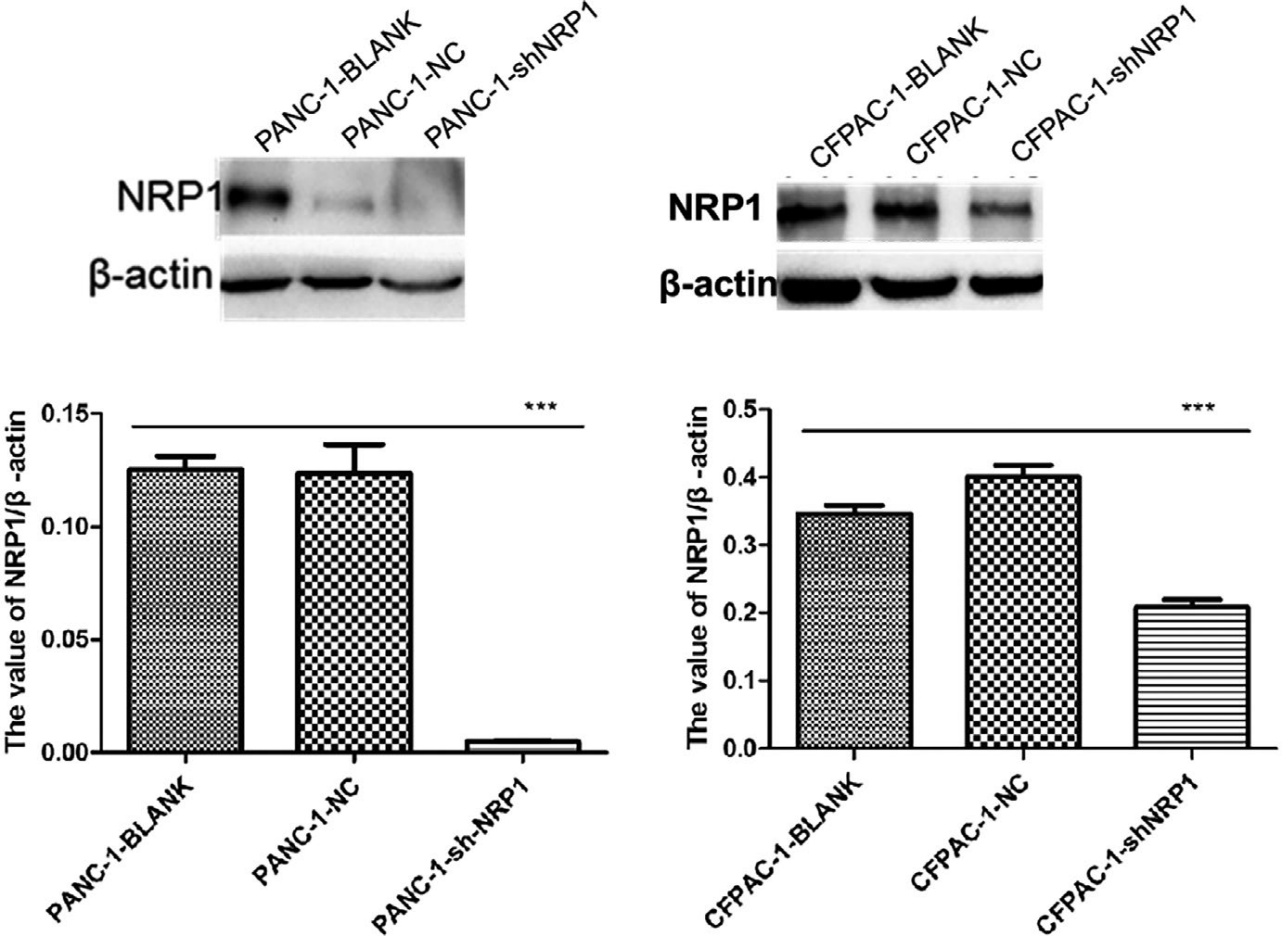
Expression of NRP1 protein in PANC‐1 and CFPAC‐1 cells after transfection, as detected with Western blot

In order to further confirm the transfection efficiency of sh1 NRP1, on the third day after transfection, the lentivirus‐transfected PANC‐1 and CFPAC‐1 cells were observed to express NRP1 green fluorescent protein (GFP), under a fluorescence microscope (Olympus Corp.). The fields of view were randomly selected to determine transfection efficiency of PACA cells under the fluorescent microscope, and take pictures of the same under fields of bright vision and fluorescence (Figure 1). Transfection efficiency > 80% at 20 multiplicity of infection (MOI) and the state of good cell growth suggested successful and stable lentivirus transfection, indicating that NRP1 sh1 RNA group may be used for subsequent experiments (Figure 2). Meanwhile, total protein from the sh1 NRP1‐transfected PANC‐1 and CFPAC‐1 cells was extracted and used to detect the expression level of NRP1 by Western blot assays. The expression level of NRP1 protein in the experimental group was remarkably reduced compared to that in the NC group (Figure 3). Based on the above findings, the sh1 NRP1 interference worked best, so the PANC‐1 and CFPAC‐1 cells transfected by sh1 NRP1 were selected for testing in the functional experiments.

### 3.3 NRP1 silencing impairs proliferation in PANC‐1 and CFPAC‐1 cell lines in vitro

**Figure 4:**
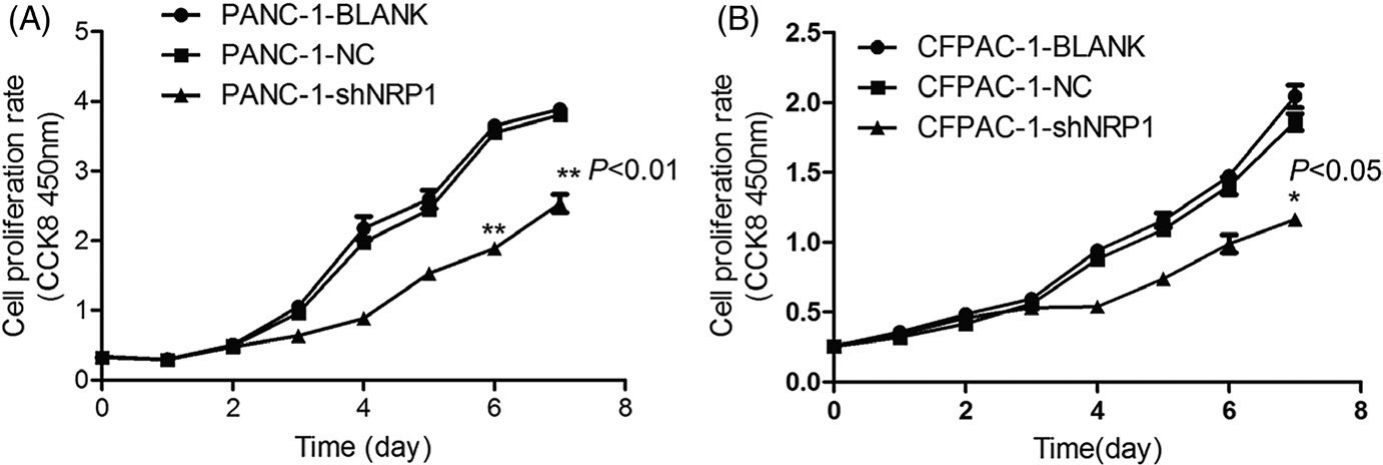
Proliferation of (A) PANC‐1 and (B) CFPAC‐1 cells after NRP1 gene silencing, as detected by CCK8 assay

In the CCK‐8 assay, the proliferation ability of NRP1 shRNA group was apparently worse than that of NC and blank groups in the PANC‐1 cells, especially in the 6 and 7 days (*P* < .01). CFPAC‐1 cells showed the same trend, and the proliferation ability of NRP1 shRNA group was apparently worse than that of NC and blank groups in the 7 day (*P* < .05), hence suggesting that the proliferation capacity of NRP1 shRNA group was apparently lower. The results revealed that eliminating NRP1 can continuously and effectively impair proliferation in PANC‐1 and CFPAC‐1 cells (Figure 4).

### 3.4 NRP1 silencing impairs invasion and migration in PANC‐1 and CFPAC‐1 cell lines in vitro

**Figure 5:**
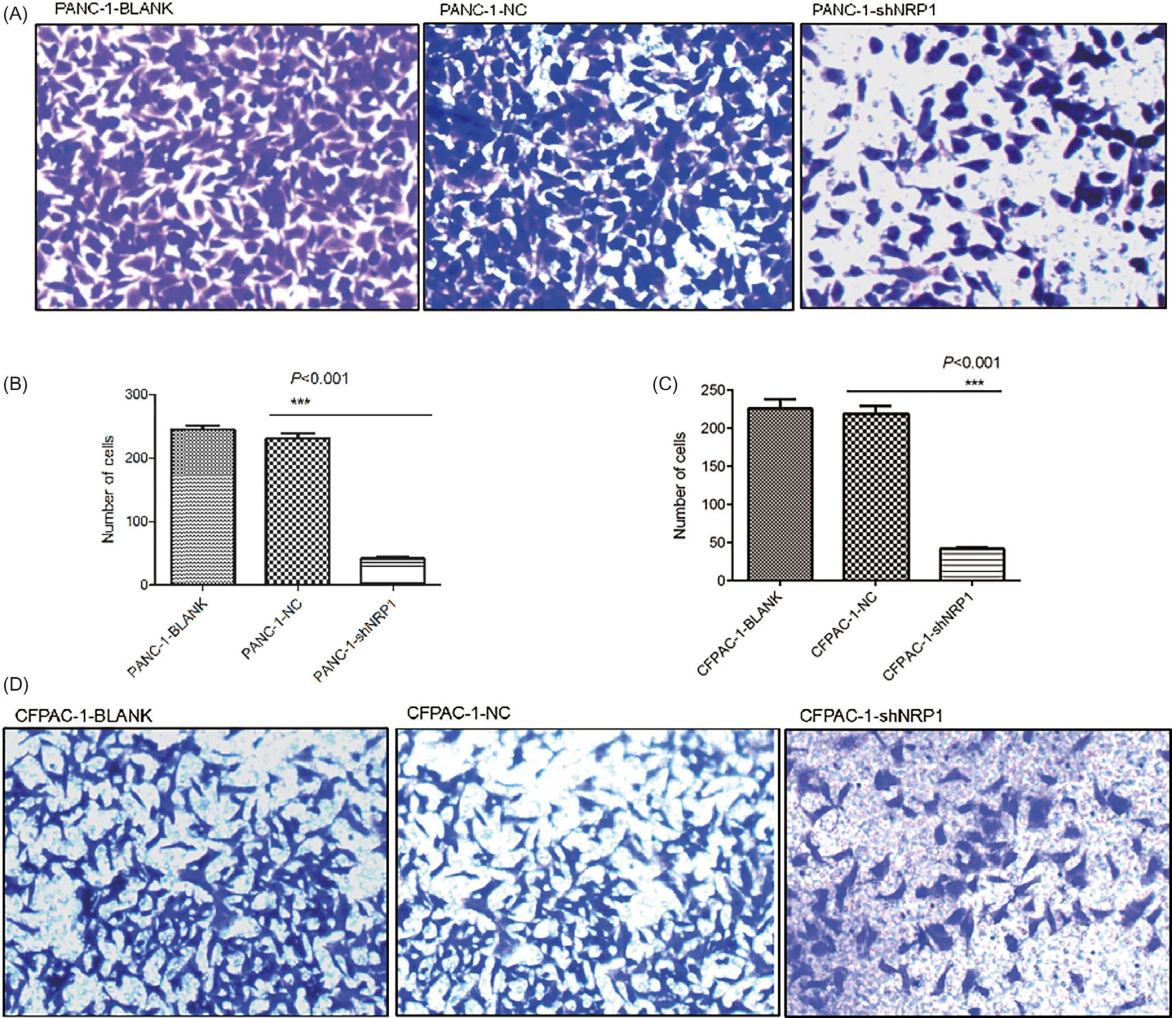
Migration of PANC‐1 and CFPAC‐1 cells after NRP1 gene silencing, as detected by transwell assay

**Figure 6:**
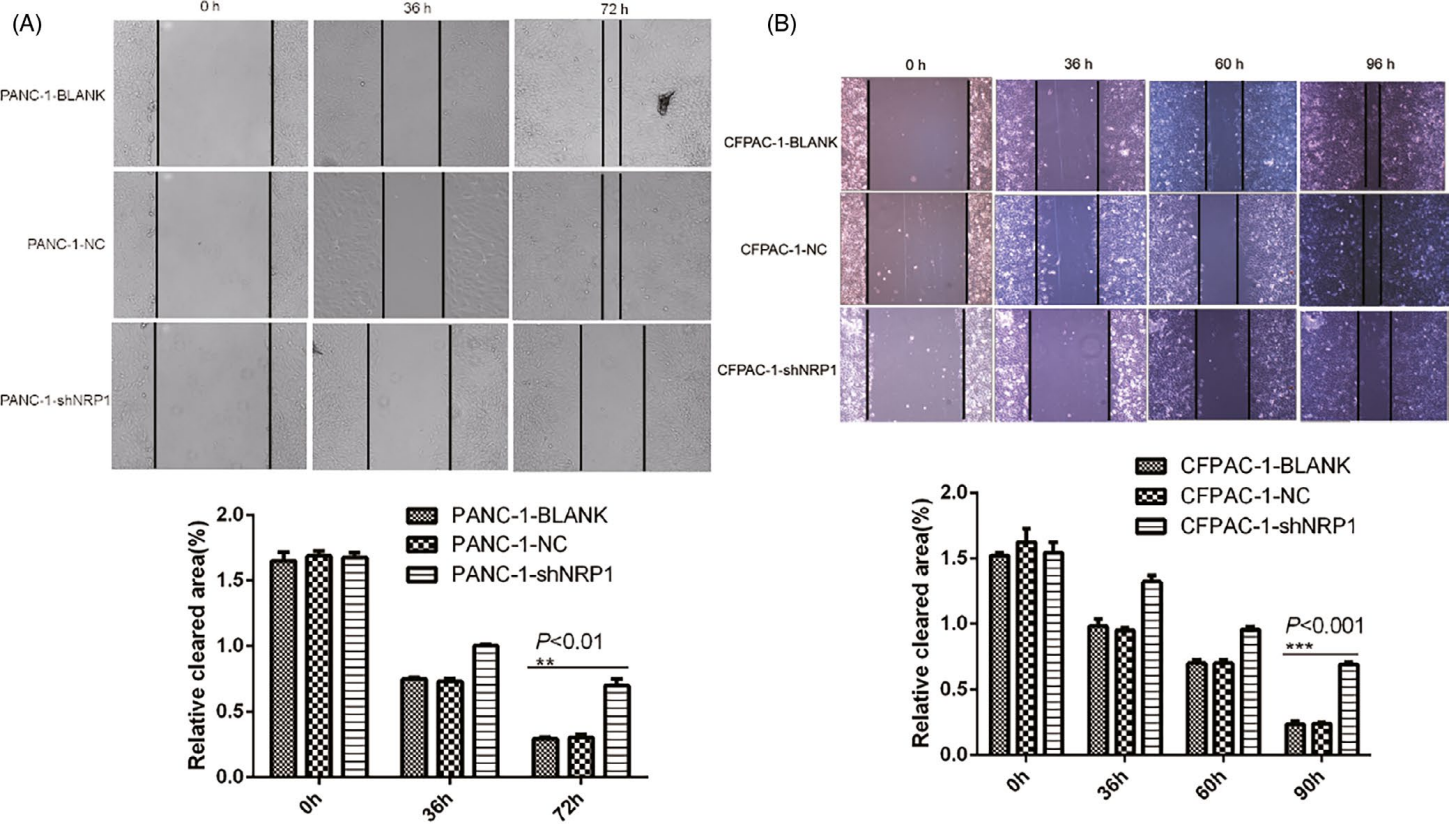
Migration of (A) PANC‐1 and (B) CFPAC‐1 cells after NRP1 gene silencing, as detected by scratch test

For the PANC‐1 transwell migration assay, the average number of transmembrane cells was 46.5 ± 3.8 in the NRP1 shRNA group, 228.4 ± 6.7 in the NC group, and 256.1 ± 5.6 in the blank group. Compared to the NC and blank groups, the number of transmembrane cells in the NRP1 shRNA group was reduced significantly (Figure 5, *P* < .001). CFPAC‐1 cells show the same trend, the average number of transmembrane cells being 42.3 ± 5.2 in the NRP1 shRNA group, 219.6 ± 4.9 in the NC group, and 223.6 ± 5.8 in the blank group. In the PANC‐1 cell wound healing assay, we measured the relative blank area of the three groups at 0, 36, and 72 hours after scratching. The difference between NRP1 shRNA group and other two groups (NC group and blank group) was confirmed to be statistically significant at 72 hours (Figure 6A, *P < *.01). In the CFPNC‐1 cell, the time is 0, 36, 60, and 90 hours, and the difference between NRP1 shRNA group and other two groups (NC group and blank group) was confirmed to be statistically significant at 90 hours (Figure 6B, *P* < .001). Results of the above two assays supported each other, and revealed that silencing NRP1 can effectively impair invasion and migration in PANC‐1 and CFPAC‐1 cells.

## 4 DISCUSSION

Currently, the expression and role of NRP1 in tumors have attracted the attention of researchers. Much evidence has confirmed NRP1 is overexpression in a range of human malignant tumors and is also closely related to the degree of malignancy.^21^, ^22^, ^23^ Previous published studies have indicated that NRP1 protein is highly expressed in human PACA tissues, and it is closely related to angiogenesis, tumor stage, and poor postoperative overall survival.^24^ Although there are some reports about the relationship between NRP1 and PACA, research related to proliferation and migration of NRP1 in PACA has been rarely reported.^24^, ^25^, ^26^, ^27^ Therefore, we were interested in the effect of NRP1 on PACA cells function.

To confirm the effect of NRP1 in human PACA cells, RNA interference (RNAi) technology was used to explore the relationship between silencing NRP1 and the ability of proliferation, invasion, and migration in PANC‐1 and CFPAC‐1 cells. RNAi can not only inhibit the expression of certain oncogenes in tumor cells, but also further inhibit tumor proliferation and metastasis thereby aiding the prevention and treatment of cancer with high efficiency, high specificity, and low toxicity features.^28^, ^29^ We silenced NRP1 gene expression in the present study using lentivirus transfection technology. The expression levels of NRP1 mRNA and protein in PANC‐1 and CFPAC‐1 cells were significantly decreased after shRNA lentiviral vector transfection, hence verifying good interference effect of lentiviral vector. Results showed that PANC‐1 and CFPAC‐1 cells proliferation, invasion, and migration ability of the virus transfected group was significantly reduced compared to that in the NC and blank groups. Based on these findings, we think that NRP1, as a potential promoting factor, plays a significant role in the proliferation, invasion, and migration of PACA cells, and may promote the occurrence, development, and metastasis of PACA.

Previous studies have shown that the silencing of NRP1 in pancreatic ductal adenocarcinoma has multiple cellular and molecular antitumor effects. Although different methods are used, the experimental results are consistent with our results that NRP1 silencing can inhibit the proliferation and migration of pancreatic cancer cells. In addition, we discussed the CFPAC‐1 cell line and to determine cell invasion and cell migration capability, we performed transwell assay and wound healing assay.^17^ This study provided a reference for exploring NRP1 gene further for molecular mechanisms of tumor invasion and metastasis, and suggests an important candidate target and new direction for the treatment of PACA. Further research should be undertaken to confirm the effect of overexpression of NRP1 gene on the function of human PACA cells. In future, to develop a full picture of NRP1, more research will be needed, such as in vivo animal experiments. In addition, research needs to be focused on NRP1‐specific function in the occurrence and progression of PACA, mechanism of influence of NRP1 on the proliferation, migration and metastasis of PACA, and contribution of NRP1 to the development of PACA signaling pathway.^30^, ^31^

## 5 CONCLUSIONS

This study has shown that NRP1‐shRNA lentiviral interference vectors can effectively decrease NRP1 gene expression in PANC‐1 and CFPAC‐1 cells, thereby inhibiting cell proliferation and migration. These findings contribute in several ways to our understanding of NRP1 and provide a basis for finding a valuable potential therapeutic target for PACA therapy.

## CONFLICT OF INTEREST

The authors declare that there is no conflict of interest with respect to the research, authorship, and publication of this article.

## AUTHOR CONTRIBUTIONS

Li‐hong He, Yong‐lin He, Yue Kang, and Ling‐yun Wang conceived and designed the study, performed the experiments, and wrote the article; Wen‐hang Zuo and Huan Xue analyzed the data, and designed the tables and figures; Yun‐liang Zhang and Yong Meng discussed results and advised during the completion of the study. All authors read and approved the final article.

## CONSENT FOR PUBLICATION

Not applicable.

## Supporting information

Sup info

## Data Availability

The analyzed data and materials during the study can be obtained from the corresponding author on reasonable request. E‐mail address: doctor1909@163.com.
