# Supplementary material for: Neuropilin1 silencing impairs the proliferation and migration of cells in pancreatic cancer
Source: J Clin Lab Anal. 2020 May 30;34(9):e23394. doi: 10.1002/jcla.23394 (PMC7521280; doi:10.1002/jcla.23394)
Supplement: Supplementary file 1 — Sup info [file JCLA-34-e23394-s001.docx]

**Supplementary Information**

**1 Quantitative real****-time polymerase chain reaction (****qRT-PCR)**

Total RNA from the cell lines were extracted using TRIzol reagent (TaKaRa, Dalian, China), according to the manufacturer's instructions. RNA concentration was detected by Nano Drop 1000 (Thermo Fisher Scientific, USA), and complementary DNA was synthesized with a Prime Script RT reagent kit (Takara, Japan) using 2 µg RNA. SYBR-Green dye (TaKaRa) and Corbett Rotor-Gene 3000 thermocycler were used to perform the qRT‑PCR reaction, according to the manufacturer’s protocol. The amplification conditions of quantitative qRT-PCR were set as follows: 95 ˚C for 30 s, 95 ˚C for 5 s, 60 ˚C for 34 s, 95 ˚C for 15 s and a total of 40 cycles. The experiments were repeated in triplicate. Analysis of the Cycle threshold (Ct) value suggested the difference between mRNA expression levels of NRP1 gene in each group.

**2** **Protein extraction and western blot (WB) analysis**

Total protein was extracted using ice-cold lysis buffer (50 mM Tris, pH 7.4, 150 mM NaCl, 1 % SDS, 1 mM EDTA, 1 % NP-40), containing 1 mM protein inhibitor and 1 mM PMSF, for 30 mi on ice. The lysates were centrifuged at a speed of 3000 g at 4 ˚C for 15 min and the supernatants collected. Protein concentration was determined using the BCA protein assay. Protein samples (50 µg/lane) were separated by electrophoresis on 10 % SDS-polyacrylamide gel electrophoresis (SDS‑PAGE) and then transferred onto polyvinylidene difluoride (PVDF) membrane (Millipore, Billerica, MA) in a wet transfer system (Bio-Rad). PVDF membranes were blocked with 2 % BSA for 1 h. Membranes were incubated with primary antibody (1:1000 dilution for NRP1 ab25998 Abcam UK;1:1000 dilution for β-actin ab8227 Abcam UK) overnight at 4 ˚C. The membranes were then incubated with the corresponding secondary antibody (1:4000 dilution, Santa Cruz Biotech, USA) for 1 h at room temperature. The immunoreactive bands were visualized using enhanced chemiluminescence reagent (Thermo, Israel), the relative protein expression was normalized to the β-actin levels and the software of Image-pro plus 6.0 (Media Cybernetics, USA) used for quantification.

**3** **Cell proliferation, invasion and migration assays.**

For the CCK-8 assay, about one thousand viable cells per hole were placed into 96-well plates in a final volume of 100 µl/well. Every 24 h, 10 µl of CCK-8 solution was added to each well, and the plate was further incubated for 2 h at 37 ˚C. Absorbance at 450 nm was quantitated using a microplate reader. The experiment was performed for 7 days, and a cell growth curve was drawn with the experimental data.

The transwell assay was performed using a 24-well plate. A transwell chamber with a polycarbonate membrane filter (Corning) was placed in the 24-well plate, pore size of the filter membrane being 8 µm. First, cells (1×10^4^/chamber) with 200 µl serum-free medium were added to the upper compartment, and 800 µl RPMI 1640 medium containing 30 % FBS was added to the lower compartment, and incubated for 48 h at 37 °C. After incubation, the cells in the upper chamber migrated to the lower surface of the membrane. The cells on the upper membrane were removed carefully with a cotton tip; the penetrated polycarbonic membrane was fixed with 4 % neutral poly formaldehyde for 30 min, and stained with 0.1% crystal violet for 30 min. The number of migrated cells was counted in 6 randomly selected fields under an inverted microscope. Independent experiments were performed in triplicate.

For the wound healing assay, tumor cells were seeded in 6-well plates at a density and incubated till 70–80 % confluence as a monolayer. A cell-free straight line was scratched at the center of the well with a sterile 1000-μl pipette tip. Another straight line was scratched perpendicular to the first line to produce a cross-shaped cellular gap in each well. The cells were subsequently washed twice with PBS and refreshed with medium containing 10 % FBS. The scratch was photographed under a fluorescence microscope every 24 h until it was filled with cells. Digital images of the cell gap were captured at different time points under the microscope.
